# Graph Analysis of TMS–EEG Connectivity Reveals Hemispheric Differences following Occipital Stimulation

**DOI:** 10.3390/s23218833

**Published:** 2023-10-30

**Authors:** Ilaria Siviero, Davide Bonfanti, Gloria Menegaz, Silvia Savazzi, Chiara Mazzi, Silvia Francesca Storti

**Affiliations:** 1 Department of Computer Science, University of Verona, Strada Le Grazie 15, 37134 Verona, Italy; ilaria.siviero@univr.it; 2 Perception and Awareness (PandA) Lab., Department of Neuroscience, Biomedicine and Movement Science, University of Verona, Piazzale Ludovico Antonio Scuro 10, 37124 Verona, Italy; davide.bonfanti@univr.it (D.B.); silvia.savazzi@univr.it (S.S.); chiara.mazzi@univr.it (C.M.); 3 Department of Engineering for Innovation Medicine, University of Verona, Strada Le Grazie 15, 37134 Verona, Italy; gloria.menegaz@univr.it

**Keywords:** TMS–EEG, occipital stimulation, TEP, brain functional connectivity, graph analysis

## Abstract

(1) Background: Transcranial magnetic stimulation combined with electroencephalography (TMS–EEG) provides a unique opportunity to investigate brain connectivity. However, possible hemispheric asymmetries in signal propagation dynamics following occipital TMS have not been investigated. (2) Methods: Eighteen healthy participants underwent occipital single-pulse TMS at two different EEG sites, corresponding to early visual areas. We used a state-of-the-art Bayesian estimation approach to accurately estimate TMS-evoked potentials (TEPs) from EEG data, which has not been previously used in this context. To capture the rapid dynamics of information flow patterns, we implemented a self-tuning optimized Kalman (STOK) filter in conjunction with the information partial directed coherence (iPDC) measure, enabling us to derive time-varying connectivity matrices. Subsequently, graph analysis was conducted to assess key network properties, providing insight into the overall network organization of the brain network. (3) Results: Our findings revealed distinct lateralized effects on effective brain connectivity and graph networks after TMS stimulation, with left stimulation facilitating enhanced communication between contralateral frontal regions and right stimulation promoting increased intra-hemispheric ipsilateral connectivity, as evidenced by statistical test (*p* < 0.001). (4) Conclusions: The identified hemispheric differences in terms of connectivity provide novel insights into brain networks involved in visual information processing, revealing the hemispheric specificity of neural responses to occipital stimulation.

## 1. Introduction

Vision is the most frequently used sensory modality in everyday life and understanding what we see around us is conditional upon successful navigation of the external environment. This represents a challenge that our brain is continuously called to face. Within the domain of visual information processing, human brain hemispheres have long been considered functionally comparable. However, a growing body of evidence suggests the existence of a hemispheric specialization, according to which the two hemispheres contribute to visual information processing in a complementary manner, where the left hemisphere is assumed to prevail over the right one in processing local details of visual stimuli, and the right hemisphere is assumed to prevail in handling global information [1]. Characterizing the dynamics of cortical activation over time and disentangling the nature of the contribution of different brain areas to visual perception may provide potential better insight into diagnostic and prognostic markers. In addition, a better knowledge of how the involved brain areas are connected could also be crucial in the search for the recovery mechanisms after brain injuries, thus becoming of great relevance in different clinical applications [2,3], such as assisting brain surgery by minimizing damage to critical brain regions or devising personalized treatments for patients affected by visual field defects.

### 1.1. Brain Stimulation and Evoked Potentials

The investigation of human brain activity involves various brain stimulation or neuromodulation techniques [4,5,6], which are typically non-invasive, and limited contraindications and side effects can occur. Among these, transcranial magnetic stimulation is widely adopted for studying cognitive functions for research purposes [4]. The effectiveness of this technology has been validated in clinical applications for treating neurological conditions [7,8], but also in neuropsychiatry [9] and psychology [10]. In principle, TMS stimulates the brain through a brief high-intensity magnetic field applied to the surface of the scalp [11]. TMS is capable of evoking cortical activity without causing significant pain. It offers the flexibility to stimulate different brain areas by adjusting parameters, such as pulse intensity, inter-pulse interval, coil type, coil orientation, and placement. Several stimulation protocols have been developed, including, single-pulse stimulation and paired-pulse TMS [12]. By inducing local neuron depolarization and eliciting action potentials, single-pulse TMS is valuable for causal mapping of neural networks, investigating motor and other non-motor systems, as well as the investigation of cortical excitability, inhibition, and plasticity [13,14].

Extensive research has been conducted on the TMS stimulation of the primary motor cortex (M1) and the dorsolateral prefrontal cortex (DLPFC) resulting in well-understood and reproducible peak responses [15]. On the other hand, the temporo-occipital cortex, occipital, and cerebellar areas have been less investigated [16,17]. In particular, when TMS is applied to the occipital cortex, it can induce visual sensations without light entering the eyes, the so-called ‘phosphene’ [18,19].

TMS-evoked potentials (TEPs) can be measured when combining TMS with electroencephalography (EEG), a non-invasive technique for monitoring brain activity with high temporal resolution by placing the electrodes on the scalp. Thanks to EEG, complex neural processes can be detected to evaluate the neurophysiological function of the brain at rest or during specific tasks or stimulation paradigms. Indeed, TEPs are complex waveforms with multiple peaks occurring within hundreds of milliseconds after the TMS pulse [15,20]. These waveforms consist of positive (P) and negative (N) deflections that reflect a spatiotemporal superposition of excitatory and inhibitory postsynaptic potentials. A primary response is typically associated with a brain activity underlying the target area and a secondary response is related to the close or distant connected brain regions. Consequently, TEPs provide a means to investigate the excitability and connectivity of the cortex [15].

Despite the great potential of TMS–EEG, the extraction of reliable TEPs is hampered by several instrumental and physiological artifacts, and obtaining a ‘true’ noise-free response is challenging [21]. The accuracy of the estimated TEPs also depends on the number of trials, which directly impacts the signal-to-noise ratio (SNR). TEPs are embedded in a background of spontaneous EEG activity. Typically, TEPs are determined by conventional averaging (CA) which is performed by averaging the EEG recordings (sweeps or trials) acquired after *N* stimuli. However, CA assumes that the TEP does not vary with the succession of stimuli and the EEG noise remains stationary during the recordings. However, the EEG signal is not stationary, except in short intervals typically lasting 1–2 s. Additionally, the CA does not leverage any “a priori” knowledge of TEP and EEG, thus it fails to exploit potentially valuable information and it cannot detect any possible TEP changes that may occur during EEG recording. To overcome these limitations, several approaches have been proposed, including weighted averaging techniques, optimal filtering methods, and single-sweep approaches [22]. However, the absence of a “ground truth” against which the acquired signal can be compared poses challenges in determining the most effective approaches to obtain a signal free from artifacts. In the literature, alternative methods have been proposed to improve SNR, especially when the number of trials is limited. These methods perform a single trial analysis based on “a priori” knowledge within Bayesian approaches [23,24], time-frequency techniques [25], and Shannon entropy [26].

### 1.2. Brain Functional and Effective Connectivity

The co-registration of TMS–EEG represents a necessary step for the quantitative assessment of the cortical excitability and brain connectivity of the human cortex [20,27]. In particular, TMS–EEG offers the possibility of monitoring the temporal dynamics of brain connectivity [28,29,30,31]. Over time, the techniques used for analyzing, extracting, and interpreting the brain networks have undergone rapid advancements, from simple assessment of temporal correlation among multiple brain regions to more sophisticated models that incorporate temporal variability and causal non-linear relationships [32]. In this domain, two well-known and established approaches are functional (FC) and effective connectivity (EC).

FC is typically derived from bivariate or multivariate measures and reflects statistical dependencies between different units of the brain. Bivariate synchrony measures capture the level of synchronization between pairs of signals by using either linear measures, such as correlation or coherence, or nonlinear dependencies, such as phase values or mutual information [33]. Conversely, multivariate synchrony approaches aim to assign a singular numerical value that quantifies the degree of synchronization within a group of signals. Some examples are the omega estimator, the S-estimator, and its extension based on the Rényi entropy [34,35]. Thus, FC measures the synchronism between signals allowing the identification of a certain degree of association between different processes. However, it does not give any measure of causality, namely understanding which of the two signals pilots the other.

EC is defined as the influence that one neural system exerts over another through causal or non-causal effects [36]. It focuses on understanding how information flows are integrated within the brain network, delving into specific pathways and mechanisms of neural activity. Specifically, the EC is a directed measure able to distinguish the incoming and outgoing information flows, unlike the FC, which is insensitive to the direction. The estimation of EC has been addressed through several strategies, which can be broadly classified into model-based and model-free techniques. Model-based approaches require the specification of structural parameters, whereas model-free methods, such as Granger causality (GC) or transfer entropy, do not require such specifications. GC is one of the most common methods aiming to statistically determine causality between variables using a linear vector autoregressive (VAR) model. Directed transfer function (DTF) and partial directed coherence (PDC) are two frequency domain representations of the GC [37]. DTF determines the directional influences across the components in a multivariate framework [38]. On the contrary, PDC quantifies the direct pathway from variable *j* to variable *i*, excluding the influence of other processes [37]. PDC offers an enhanced estimation of the directional information flow between different regions of the brain compared to DTF. Indeed, it takes into account both the direct and indirect influences among variables, leading to a more comprehensive understanding of the underlying connectivity patterns [39]. The information partial directed coherence (iPDC) is another spectral measure that considers the effects of signal size when evaluating connection strength [40].

Traditionally, brain connectivity studies have focused on static or averaged connectivity patterns, assuming that the brain operates with fixed connectivity. Static connectivity networks are obtained from the above-mentioned methods, thus they do not explain the temporal variability of the brain connections [33]. However, the time course of functional brain connectivity plays an important role in detecting the propagation of neuronal pathways and is a crucial aspect of understanding how the brain works, as it is well known that the brain’s functional architecture varies over time [41]. For this reason, other methods have been recently proposed to infer the time-variability of the network [42]. Methods used to study time-varying brain connectivity include dynamic causal modeling (DCM), sliding window approaches [43], and time-frequency analysis such as short-time DTF [39]. Markov model-based frameworks [44] and Kalman filter-based approaches [39,45] are other examples to model the time-varying brain networks. Dynamic functional connectivity (dFC), also known as time-varying connectivity, has been primarily studied in the domain of EEG or epilepsy [46] and functional magnetic resonance imaging (fMRI) [47], but not exclusively. In particular, to obtain a network that is robust from noise, Pascucci et al. proposed a self-tuning optimized Kalman (STOK) filter [48]. Its purpose was to ensure accurate tracking of networks, precise temporal localization, and robustness to noise by incorporating a self-adjusting memory decay and a recursive regularization technique. The STOK filter was tested both in silico and with real data, and its performance was found to be better than the one obtained by employing the traditional Kalman filter.

These methodologies provide insights into the dynamical organization of the brain and its underlying processes in response to cognitive demands, task requirements, or neurological conditions. Connectivity-based analysis has been proposed for many TMS–EEG studies, with applications ranging from pathological conditions, such as Alzheimer’s disease [49] to normal brain functioning [50]. Other studies provided tools for consciousness classification (e.g., vegetative state and minimally conscious state). For example, Rosanova et al. proposed a reliable approach to detect the recovery of consciousness by analyzing the differences between hemispheres using EC models [2]. Similar findings were reported by Ragazzoni et al. in an independent TMS–EEG study [51]. Moreover, several studies investigated brain connectivity and the differences in information flow at rest versus the execution of cognitive tasks [52,53]. In the context of occipital TMS, brain connectivity analysis has already been used to examine its network-level effects, but the predominant focus in the literature has mainly been on attention protocols [54] and visual adaptation [55], leaving other potential applications relatively unexplored.

### 1.3. Brain Graph Networks

Graph theory has emerged as a powerful framework for studying brain connectivity, representing the brain as a network of interconnected nodes (or vertices), which correspond to neurons or brain regions, and links (or edges), which represent the relationships between those regions [56]. Both local (functional segregation) and global (functional integration) properties can be assessed through different measures of graphs. For example, centrality, clustering, modularity, and connectivity patterns can be obtained to understand the importance of specific nodes or regions, the presence of distinct functional modules, and the overall integration of information. Graph theory has found extensive use in neuroscience, particularly in studies using neuroimaging techniques, like fMRI and positron emission tomography (PET). The aim is to establish the human ’connectome’, a comprehensive map of connections within the brain. Furthermore, graph networks provide a valuable framework for studying how the activation or manipulation of specific nodes or edges affects cognitive functions, such as perception or attention, and enable the study of network dynamics. For example, Caeyenberghs et al. [57] investigated brain activity during cognitive control tasks in patients with traumatic brain injury. They studied the performance and the topological properties of the functional brain networks using a graph theoretical approach, showing increased connectivity degree and strength, as well as higher values of local efficiency. The effectiveness of the graph theory has been demonstrated in many TMS–EEG studies [53]. Most of them focused on clinical applications with the goal of examining altered networks in various physiological or pathological conditions, since TMS–EEG enables the observation of dynamic responses within the targeted region and other specific connections. However, the dynamics of brain connectivity and relative brain network pose an additional layer of complexity. In terms of analysis, it involves examining the dynamic properties of functional brain networks, such as the aforementioned strength, network efficiency, and modular organization.

### 1.4. Aim of the Work

The aim of this study is to investigate the hemispheric differences in visual information processing through a dynamic brain connectivity analysis in order to uncover the underlying mechanisms that contribute to the effects of occipital TMS. Specifically, the objective is to analyze EEG data to identify connectivity patterns resulting from single-pulse TMS delivered around the occipital EEG sites, O1 and O2 (in the left and right hemispheres, respectively). To the best of our knowledge, this issue has never been addressed in the literature and, to achieve this, we employed different steps for the processing of EEG signals. Since the aim is to explore the casual interaction among brain areas in a dynamic manner, complex models that consider temporal variability are used. In detail, a Bayesian estimation approach is used to extract the TEPs, which has been demonstrated to be powerful in obtaining a reliable estimate of evoked potentials, especially when the number of trials is limited. Then, the STOK filter is applied to track the fast changes in brain reorganization and estimate the time-varying brain EC using iPDC. Furthermore, to assess the properties and characteristics of the brain network, a graph analysis is performed employing the betweenness centrality and degree measures. Lastly, statistical tests are implemented to uncover the differences in visual processing between left and right TMS.

## 2. Materials and Methods

**Figure 1:**
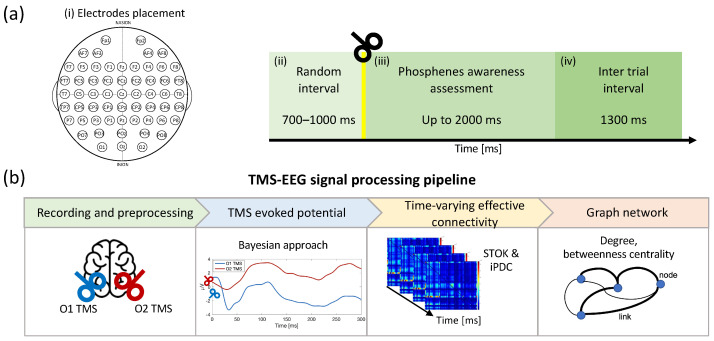
(**a**) Electrode placement and schematic representation of the experimental procedure: (i) random interval (700–1000 ms), (ii) single-pulse TMS stimulation, (iii) phosphene awareness assessment (up to 2000 ms), and (iv) inter-trial interval (1300 ms). (**b**) Signal processing pipeline for assessing the differences between hemispheres after TMS stimulation. The proposed pipeline is divided into preprocessing, TMS-evoked potential estimation, the time-varying effective connectivity calculation, and the graph network.

In this section, the TMS–EEG experimental protocol and participant recruitment are first described (Section 2.1, Section 2.2 and Section 2.3, and Figure 1a). Then, the proposed TMS–EEG signal processing framework is explained as depicted in Figure 1b. In particular, EEG data were filtered as described in Section 2.4. A state-of-the-art Bayesian approach was used to estimate the TEPs from the filtered EEG signals as described in Section 2.5. As described in Section 2.6, the STOK filter was used for tracking the dynamic behavior of the directed brain network, and the iPDC was estimated. Additional mathematical details of the Bayesian approach and the STOK filter are reported in Appendix A and Appendix B, respectively. The edges betweenness centrality and the node degree were calculated for each time-variant directed connectivity matrix as reported in Section 2.7.

### 2.1. Participants

The study recruited eighteen healthy subjects on a voluntary basis (10 females, mean age 23.66 ± 3.54), who were all right-handed and had normal or corrected-to-normal vision. All participants gave their informed consent in accordance with the Declaration of Helsinki. Data were collected according to protocols approved by our local ethics committee. To ensure safety, a questionnaire was used to screen participants for potential TMS hazards, and no contraindications were reported.

### 2.2. TMS Protocol and Experimental Protocol

Single-pulse TMS was delivered through a 70 mm figure-of-eight coil connected to a Magstim Rapid2 system (maximum output 3.5 T, the Magstim Company Limited, Whitland, UK). To prevent unnecessary neck muscle activation, the coil was positioned tangentially to the scalp surface with the handle pointing upward. Through supra-threshold phosphene induction, stimulation areas were functionally located around electrode positions O1 (left hemisphere) and O2 (right hemisphere) of the 10-10 International System. Electrodes O1 and O2 were used as a starting point around which, in a ∼2 cm^2^ area, we selected the hotspot eliciting the most consistent and clearest phosphenes while stimulating at supra-threshold intensity. Neuronavigation taking advantage of individual structural MRI images (SofTaxic, E.M.S., Bologna, Italy, and Polaris Vicra, NDI, Waterloo, ON, Canada) was used in the course of the experiment (1) during the hotspot search, to monitor that the stimulation was targeting early visual areas; (2) during the course of the experiment, to check for coil displacements larger than 2 mm accuracy threshold; and (3) to reposition the coil exactly on the hotspot after breaks between sessions. The individual phosphene threshold (PT) was set using the automatic procedure of the ’Method of Constant Stimuli’ (MOCS) [58]. After the hotspot for each stimulation site was functionally identified, the PT was evaluated using a computerized MOCS version: seven TMS intensities were used (ranging from 60% to 78% of maximum stimulator output (MSO), with an increasing step of 3%). For each of these intensities, seven pulses were delivered. Pulses from different stimulation intensities were randomly interleaved, resulting in a randomized order of stimulation intensities. After each TMS pulse, participants were asked to report any presence of phosphenes. The resulting data were then fitted with a cumulative Logistic psychometric function via a maximum likelihood criterion using the Matlab 2021b (MathWorks, Natick, MA, USA) Palamedes toolbox (http://www.palamedestoolbox.org, accessed on 1 December 2021). From the obtained function, we derived the PT intensity at which participants perceived phosphenes in 50% of trials, and this intensity was employed during the experiment as stimulation intensity.

### 2.3. Perception of Phosphenes: Protocol and Testing

Participants sat in a dark room, in front of a monitor, with their heads on a chin rest to keep their eyes aligned with the central fixation point. They were instructed to maintain their gaze on the fixation point throughout the experiment. Before the two experimental sessions, a training session was performed to test participants for the perception of phosphenes. Participants were initially debriefed about the functioning of TMS and phosphenes [58,59]. Afterward, they had to wear a cap on which the positions of electrodes O1 and O2 were labeled.

Experimenters then started administering single-pulse TMS around O1/O2 positions; after each stimulation, participants were asked if they had seen something matching phosphene characteristics in their visual field, and, if so, to give a short description of these percepts. Once participants had sufficiently adapted to darkness and experimenters were confident that they were reporting actual phosphenes, the stimulation conditions (e.g., asking participants to fixate on a different point on the screen or to close their eyes) were changed to test if participant reports still matched the expected characteristics for phosphenes. Once the procedure was completed for one hemisphere, the other one underwent testing. The criteria used to test for real phosphenes are in [59]: they appear in the visual field contralateral to the stimulated hemisphere; they follow the participants’ gaze; and they appear independently of the eyes being closed or open. After being assessed for real phosphene perception, participants underwent an MRI scan necessary for neuronavigated TMS. Two consecutive sessions were carried out and single-pulse TMS was administered to the left and right occipital cortex at PT intensity, with the order of the two stimulated sites counterbalanced across participants, while simultaneously recording EEG data. In order to mask the audible TMS click, participants were asked to wear disposable earplugs. First, a random interval with a duration of 700–1000 ms preceded the TMS pulse; the stimulation was then administered and followed by up to 2000 ms, during which participants had to report the presence or absence of a phosphene by pressing one of two keyboard keys (the ‘m’ key with the right hand for phosphene present, the ‘z’ key with left hand for phosphene absent). Finally, a 1300 ms inter-trial interval separated each trial (Figure 1a). Moreover, 360 pulses divided into 6 blocks of 60 trials each were administered during each session. Blocks were separated by a few minutes of rest, to prevent excessive fatigue in participants.

### 2.4. EEG Recording and Preprocessing

A TMS-compatible EEG equipment (BrainAmp, Brain Products GmbH, Munich, Germany) was used to record EEG activity (BrainVision Recorder, version 1.25). The recording setup involved a Fast’n East cap containing a total of 59 scalp channels, with additional electrodes used to monitor horizontal and vertical eye movements, as online reference (RM) and as ground (AFz). All of them were TMS-compatible Ag/AgCl pellet pit electrodes (Easycap GmbH, Herrsching, Germany). The electrode placement followed the extended 10-10 International System (impedance was kept below 5 kΩ).

The EEG signal data analysis was performed offline using Matlab 2021b (MathWorks, Natick, MA, USA) with the EEGLAB toolbox (version 2021.0) and the TMS–EEG signal analyzer (TESA) extension [60]. The continuous raw signal digitized at 5000 Hz was segmented into 1000 ms before and after the TMS pulse. The epochs were demeaned using the entire epoch and the TMS pulse artifact was removed from −2 to 10 ms. It was replaced with cubic interpolation to avoid ringing artifacts. Data were downsampled at 500 Hz. A first independent component analysis (ICA) round, using the ’runica’ function, was performed to remove the TMS artifacts followed by a zero-phase fourth-order Butterworth filtering between 0.1 and 100 Hz with a band-stop filter (49–51 Hz). A second ICA was used to remove blinks and muscle activity. To improve component decomposition, the interpolated data from −2 to 10 ms after the TMS pulse were replaced with constant amplitude values before each ICA, and then interpolated again afterward. The REST toolbox was used to re-reference the data to a point at infinity [61]. Then, data were low-pass filtered at 40 Hz, and bad trials were automatically rejected through the TBT toolbox [62]. Further details of the preprocessing pipeline are reported in the study by Rogasch et al. [60].

### 2.5. TEP Estimation

To estimate the TEPs, we used the Bayesian approach proposed by Sparacino et al., which is based on a sweep-by-sweep filtering strategy [23,24]. This approach incorporates a Bayesian framework by exploiting second-order statistical information on both the unknown EP and the background EEG, which varies from one sweep to another. The pre-stimulus EEG data are fitted with an autoregressive (AR) model and the unknown TEP is treated as a multiple integration of a white noise process. In each single sweep, a filtered response is obtained after the stimulus by applying a smoothing criterion. Then, the TEP is obtained by computing a weighted average of the filtered sweeps. Within the Bayesian framework, each sweep could be weighted according to its reliability, which corresponds to the estimate of the filter error. Thus, the weight assigned from each sweep is inversely proportional to the expected value of the squared norm of the filter error. For a detailed mathematical formulation of this Bayesian smoothing method please refer to Appendix A. Of note that, before computing the TEPs, the mean of pre-stimulus EEG data was subtracted to each single sweep.

In order to compare the TEPs obtained through the CA method with those obtained with Sparacino et al.’s Bayesian approach, the global mean field power (GMFP) was used. The GMFP is calculated considering the data from all recording electrodes and provides a global measure. The GMFP measure is calculated as follows:

(1) $$\begin{array}{c} \mathrm{GMFP}(t)=\sqrt{\frac{\sum_{i=1}^{K}{\left(V_{i}\left(t\right)-V_{mean}\left(t\right)\right)}^{2}}{K}} \end{array}$$

where *t* is time, *V* is the voltage at channel *i*, $V_{mean}$ is the average of the voltages in all channels, and *K* is the total number of channels. Finally, a Wilcoxon signed-rank test Bonferroni corrected, p< 0.05 was used to compare the two GMFPs for both left and right occipital TMS.

### 2.6. Time-Varying iPDC Estimated through STOK Filter

In this study, we used the STOK filter, a powerful method for investigating the information flow and causal interactions under unknown noise conditions in the cognitive domain. It embeds a self-tuning memory decay and a recursive regularization to guarantee high accuracy in network tracking, temporal precision, and robustness to noise [48]. The STOK filter is based on the time-varying multivariate autoregressive model (tvMVAR), which provides a valuable framework for describing the dynamic behavior of multiple repeated experiments involving physiological time series, such as the EEG signal. Indeed, the EEG data can be viewed as realizations $\left\{Y_{t},t=t_{1},t_{2},\ldots t_{T}\right\}$ of the same multivariate stochastic process, as follows:

(2) $$\begin{array}{c} Y_{t}=\left[\begin{array}{ccc} y_{1,t}^{\left(1\right)} & \cdots & y_{d,t}^{\left(1\right)}\\ \vdots & \ddots & \vdots \\ y_{1,t}^{\left(N\right)} & \cdots & y_{d,t}^{\left(N\right)} \end{array}\right] \end{array}$$

where *T* is the length of each time series, *N* is the total number of trials, and *d* is the number of electrodes.

In general, the tvMVAR model can be specified in the form of:

(3) $$\begin{array}{c} Y_{t}=\sum_{k=1}^{p}A_{k,t}Y_{t-k}+\epsilon_{t} \end{array}$$

where $A_{k,t}$ are the AR matrices containing the time-varying model coefficients, $\epsilon_{t}$ is the zero mean white noise with covariance matrix $\Sigma_{\epsilon }$, and *p* is the model order. The STOK filter was used to estimate the AR coefficients and the covariance matrix $\Sigma_{\epsilon }$. Then, it was used to calculate the iPDC measure. Please refer to Appendix B for the mathematical formulation of the STOK filter [48].

The iPDC is a multivariate spectral measure that compares only the directed influences between any given pair of signals (i,j). The iPDC stands out among the various approaches for extracting the EC measure by accurately considering the impact of the signal size when evaluating the connection strength [40].

By defining B(f,t) as

(4) $$\begin{array}{c} B\left(f,t\right)=I_{d}-\sum_{k=1}^{p}A_{k,t}e^{-j2\pi fk} \end{array}$$

where $A_{k,t}$ are the AR matrices estimated by the STOK filter at each time *t*, $I_{d}$ is the identity matrix and *j* is the imaginary unit; the iPDC complex function from the time series *j* to the time series *i* is obtained by:

(5) $$\begin{array}{c} {\mathrm{iPDC}}_{i\leftarrow j}\left(f,t\right)=\sigma_{\epsilon_{ii}}^{-1/2}\frac{b_{ij}\left(f,t\right)}{\sqrt{b_{j}^{H}\left(f,t\right)\Sigma_{\epsilon }^{-1}b_{j}\left(f,t\right)}} \end{array}$$

where $b_{j}\left(f,t\right)$ and $b_{ij}\left(f,t\right)$ are the *j*-th column and the (j,i)-th element of the matrix B(f,t), respectively, $\sigma_{\epsilon_{ii}}$ is the (i,i)-th element of the innovation covariance matrix $\Sigma_{\epsilon }$ [63], and the *H* in $b_{j}^{H}\left(f,t\right)$ stands for Hermitian transpose. The absolute value of iPDC${}_{i\leftarrow j}\left(f,t\right)$ is usually analyzed.

The iPDC has been used in other studies demonstrating good performance. For example, in Rubega et al., the iPDC was adopted to study brain connectivity in the visual EP of face perception and in interictal epileptiform discharges in focal epilepsy [64].

### 2.7. Graph Networks and Statistical Analysis

By representing the brain as a graph, with nodes as brain regions (in this case EEG electrodes) and edges as connections (in this case iPDC), we can quantify the network properties. Among the network measures available, degree and centrality metrics are particularly interesting in this context [53].

Specifically, the betweenness centrality is a measure of the fraction of all shortest paths in the network that pass through a given node. Degree corresponds to the number of connections of a node, proving an indicator of the node’s importance in the network [56]. Nodes with high degrees are considered influential hubs within the brain network. Differently, centrality measures the importance of a node or edge based on its position within the network. Nodes or edges with high centrality are crucial for efficient information transfer within the network. To assess the network centrality, the brain connectivity toolbox (BCT) (https://sites.google.com/site/bctnet/home?authuser=0, accessed on 1 March 2023) was used.

Differences in graph networks between two conditions were tested utilizing a Wilcoxon signed-rank test with a significance level of *p* < 0.001.

## 3. Results

The following section presents the obtained results aiming at understanding the propagation of the information in the brain network and its organization after the two different TMS brain stimulations. In Section 3.1, we compare the TEPs obtained through the CA and Sparacino et al. Bayesian approaches. Lastly, the statistically significant differences between left and right TMS are presented in terms of iPDC, edges betweenness centrality, and degree in Section 3.2.

### 3.1. TEPs Estimated through the Sparacino et al.’s Bayesian Approach

**Figure 2:**
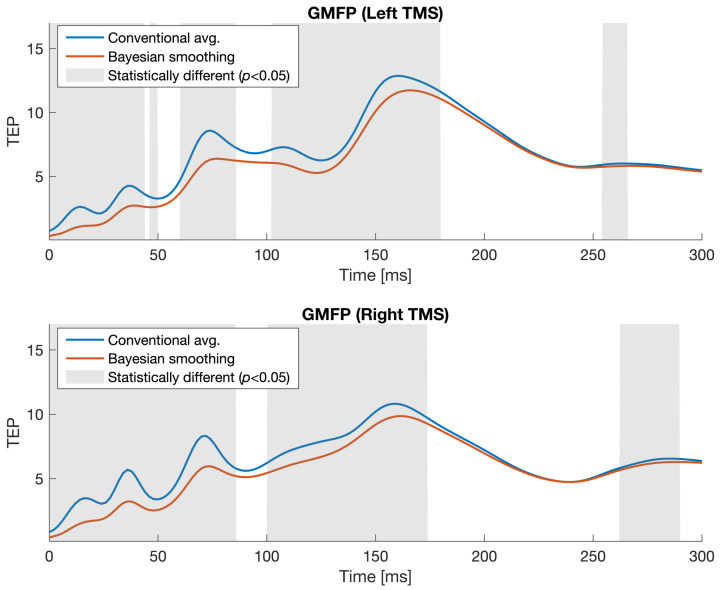
Global mean field power calculated for TEP using the conventional averaging (CA) method (blue) and the Bayesian smoothing approach (red) obtained from 360 sweeps. The upper part referred to the left TMS stimulation and the lower part to the right TMS stimulation. Statistical significance differences between the CA method and the Bayesian smoothing are indicated by gray areas (Wilcoxon signed-rank test Bonferroni corrected, p< 0.05). Time is measured in ms and the amplitude is in μV.

Figure 2 compares the GMFP for TEPs obtained using two different methods: the CA and the Bayesian approach. The upper part of the figure represents the GMFP recorded after left TMS stimulation (O1), while the lower part represents the GMFP recorded after right TMS stimulation (O2). Gray-shaded areas indicate statistically significant differences between the CA method and the Bayesian smoothing approach (Wilcoxon signed-rank test Bonferroni corrected, p< 0.05). It can be observed that the two time series appear different mainly in the first part of the GMFP after the TMS stimulation. This comparison provides valuable insights into the performance discrepancies between the two. However, it is important to note the lack of a “ground truth”, i.e., the true underlying potential in our case, which prevents further validation through TEP simulations. The Bayesian smoothing approach has been successfully validated in silico using other EP data, such as in [23]. This prior validation supports its reliability in estimating TEP characteristics.

### 3.2. Time-Varying iPDC and Graph Analysis

**Figure 3:**
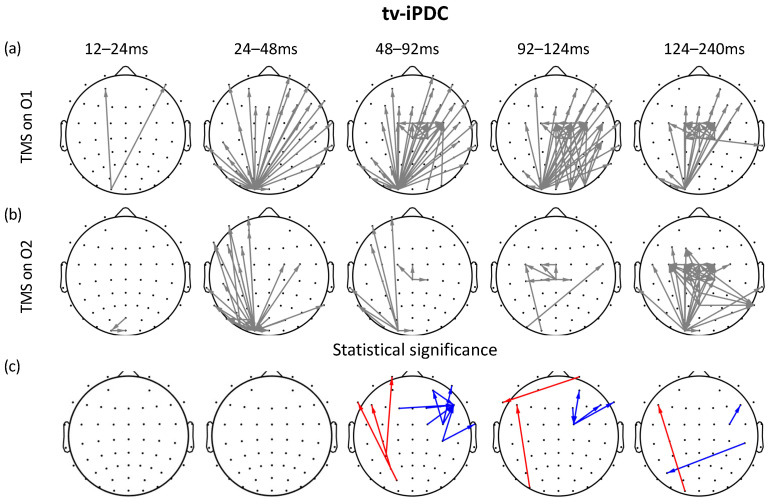
Time-varying brain connectivity analysis for stimulation sites. (**a**) TMS on O1; (**b**) TMS on O2; (**c**) statistical significance differences between conditions are indicated by arrows (Wilcoxon signed-rank test uncorrected p< 0.001). Red arrows indicate stronger connections following right stimulation (O2) than left stimulation (O1). By contrast, blue arrows indicate stronger connections following left stimulation (O1) than right stimulation (O2).

Brain EC was investigated through time-varying iPDC analysis. Figure 3 illustrates the results of the group analysis for the two stimulation sites. Figure 3a represents the connectivity analysis for the left stimulation at site O1, while Figure 3b depicts the connectivity analysis for the right stimulation at site O2. Each topoplot shows the directed connections between brain channels in five different time windows, which are 12–24 ms, 24–48 ms, 48–92 ms, 92–124 ms, and 124–240 ms. These time windows were selected around the peaks of the GMFP (Figure 2). An empirical threshold, expressed as a fixed percentage (50%) of the max value, was applied to the topographic maps for the O1 and O2 stimulations for visualization purposes, highlighting the strongest connections. To compare the differences between the two hemispheres, the electrodes and their corresponding connectivity for the right TMS stimulation were flipped from right to left (so that the two stimulation sites were overlapped). The arrows in Figure 3c indicate statistically significant differences between the two conditions (Wilcoxon signed-rank test uncorrected p< 0.001). These findings provide insights into the dynamic patterns of brain connectivity associated with the different stimulation sites. It is strongly visible that left occipital TMS produces a scalp activation that propagates more along contralateral channels than right occipital TMS (see Figure 3a,b). The statistical analysis reveals a divergence between the two stimulations during the time window spanning from 48 to 92 ms, particularly in the frontal regions of the contralateral hemisphere and between the occipital and frontal electrodes in the same hemisphere. The connections in Figure 3c appear positive when the statistical test detects that the stimulation at O1 is stronger than at O2 (see the blue arrows in Figure 3c). Conversely, the connections are negative when the test detects that the stimulation at O2 is more intense than at O1 (see the red arrows in Figure 3c).

**Figure 4:**
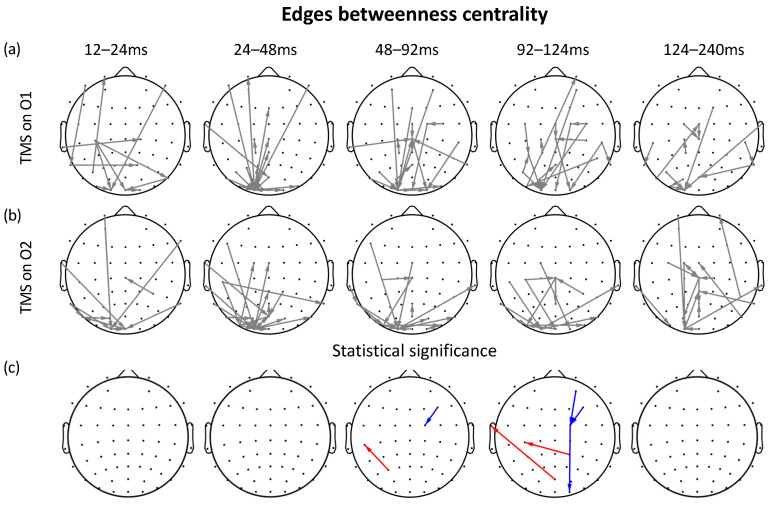
Time-varying edges betweenness centrality for stimulation sites. (**a**) Left (O1) TMS stimulation; (**b**) right (O2) TMS stimulation; (**c**) statistical significance differences between conditions are indicated by arrows (Wilcoxon signed-rank test uncorrected p< 0.001). Red arrows indicate stronger values of the edges following right stimulation (O2) than left stimulation (O1). By contrast, blue arrows indicate stronger values of the edge following left stimulation (O1) than right stimulation (O2).

Betweenness centrality analysis of time-varying edges was performed to investigate the importance of edges (connectivity links) between stimulation sites. Figure 4 shows the results of this analysis for both left (O1) and right (O2) TMS stimulation. Figure 4a represents the time-varying edges betweenness centrality analysis for left TMS, while Figure 4b corresponds to the analysis for right TMS. The arrows in Figure 4c indicate statistically significant differences between the conditions (Wilcoxon signed-rank test uncorrected, p< 0.001). In this case, the significant EC connections are more restricted and occur within the time windows of 48–92 ms and 92–124 ms. However, positive edges (O1 > O2) are observed in the contralateral channels to the stimulation, while negative ones (O2 > O1) are circumscribed to ipsilateral channels. This result suggests that these connections play a central role in information transmission.

**Figure 5:**
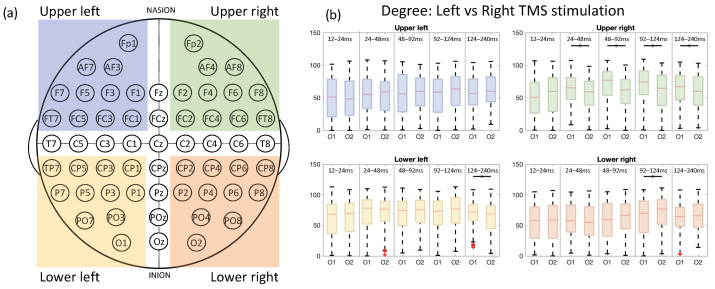
(**a**) Electrode placement using the international 10-10 system covering the ipsilateral frontal channels (highlighted in blue), contralateral frontal channels (highlighted in green), ipsilateral occipital channels (highlighted in yellow), and contralateral occipital channels (highlighted in orange). (**b**) Degree metric of graph networks in response to left vs. right TMS. Statistical significant differences between conditions are indicated by asterisks above boxplots (Wilcoxon signed-rank test uncorrected p< 0.001).

Figure 5 shows the degree metric of the graph network for the two stimulation sites. Since the results depicted in Figure 3 and Figure 4 showed stronger connections in the ipsilateral hemisphere and the frontal regions of the contralateral hemisphere over time, here, the brain was divided into four macro areas, i.e., ipsilateral (to the TMS stimulation) occipital, ipsilateral frontal, contralateral occipital, and contralateral frontal. These areas are visually represented as light blue, green, yellow, and orange, respectively. For example, the ipsilateral frontal area (light blue) includes the electrodes Fp1, AF7, AF3, F7, F5, F3, F1, FT7, FC5, FC3, and FC1. All degree values within each specific area were grouped and plotted for every time window by using a boxplot. Statistical differences between the left and right TMS stimulation were depicted by black asterisks above boxplots in each time window. This analysis revealed significant differences in degrees between left and right TMS conditions in the contralateral frontal area from 24 ms to 240 ms, in the ipsilateral occipital area from 124 to 240 ms, and the contralateral occipital area from 92 to 124 ms, as assessed by statistical testing (Wilcoxon signed-rank test uncorrected p< 0.001).

## 4. Discussion

Due to excellent temporal resolution, TMS–EEG has emerged as a powerful technique to characterize TMS-induced connectivity from a functional perspective. Indeed, EEG allows us to trace the trans-synaptic spread of activation to remote but interconnected brain regions as a result of the local activation triggered by the magnetic stimulation of the targeted cortical area. To the best of our knowledge, the study of hemispheric differences within the visual domain has never been addressed by applying TMS to the early visual cortex and concurrently recording EEG signals. For this reason, we aimed at investigating physiological hemispheric differences concerning the spatiotemporal dynamics of signal propagation during occipital stimulation. To do this, a time-varying signal processing pipeline was proposed based on existing techniques in order to understand how the brain areas are involved during the occipital TMS–EEG stimulation.

### 4.1. Mapping the Asymmetries of Functional Connectivity within Visual Networks after Left and Right Occipital TMS

Our findings show that, by targeting the left early visual cortex (O1), the resulting TMS-induced signal propagation pattern involved more contralateral channels than the right visual cortex stimulation, especially regarding frontal electrodes. Conversely, the stimulation of the right early visual cortex (O2) elicited increased intra-hemispheric connectivity, especially affecting occipital and frontal areas ipsilateral to the (right) stimulated site. These results point towards a complex effect on visual signal propagation, by triggering side-specific spatiotemporal dynamics, providing further support to the hypothesis that a functional left–right hemispheric asymmetry exists for low-level functions as well. Since Broca’s work on aphasia paved the way for modern neuropsychology by investigating patients presenting with lateralized focused brain lesions [65,66], the human brain hemispheres have no longer been considered functionally equivalent. Traditionally, however, this lateralization was always thought to selectively concern high-level functions, such as language and attention. The new data presented here prove an asymmetry across the two hemispheres in visual information processing by highlighting that the right hemisphere is the most dominant for visual function, at least under these circumstances. Indeed, when stimulating the right hemisphere, brain connectivity is circumscribed to ipsilateral channels, and no significant involvement of contralateral networks was found. This right-hemispheric specialization for visual function is supported, to date, by many sources of converging evidence, including behavioral and TMS studies [67,68], neuropsychological evidence [69], electrophysiological findings [70], and neuroimaging results [71,72]. Interestingly, evidence from a previous TMS-fMRI study [71] highlights a key role of the right frontal cortex in line with our results, revealing that frontal or parietal TMS administered to the right rather than to the left hemisphere exerts a stronger influence upon the visual cortices via back-projection. Intriguingly, our data show that such an effect can also occur the other way around, namely stimulating the visual cortex and monitoring the signal propagation towards right frontal areas, in a bottom-up fashion. Moreover, our data possibly explain right-hemisphere lesions in frontal or parietal areas that lead to deficits affecting the visual domain. Our results can also corroborate previously cited evidence showing that the left hemisphere prevails in processing local visual details, while the right hemisphere prevails in handling global visual information [67]. Indeed, participants in our study were not asked to actively process any local details of a visual stimulus, and the TMS-induced signal propagation pattern following the left hemisphere stimulation mostly involved contralateral channels, pointing to the need for a right hemisphere involvement. Differences in the brain response between the two hemispheres after single-pulse TMS delivered to the temporo-occipital and dorsolateral prefrontal brain areas were also found by Jarczok et al. [73]. The findings of this study show that TEPs are lateralized towards the stimulated hemisphere.

Finally, in the context of TMS–EEG, a recent study focused on investigating how attention promotes the gating of information from the sending area to the receiving areas, achieved through dynamic changes in EC [54]. To probe the EC and cortical excitability modulated by covertly shifted attention, TMS was used to perturb the right retinotopic visual cortex in relation to attended and unattended locations. Stimulation of the contralateral visual hemisphere resulted in stronger EEG responses and increased connectivity compared to the ipsilateral hemisphere. The time-delayed phase locking value (tdPLV) was used to estimate the effective connectivities between O2 and all other electrodes. However, while this allowed the evaluation of the inter-area phase synchronization, all the other connections were not assessed.

In the long term, understanding whether—and to what extent—visual network connectivity is functionally lateralized in the human brain is of paramount importance for several reasons. Among others, it can have clinical implications [2], thereby helping to predict the outcome of brain surgery when resection involves visually responsive areas. At the same time, being aware of connectivity patterns characterizing the healthy brain can also help to develop and customize rehabilitation interventions for visually impaired patients as a function of the affected hemisphere.

### 4.2. Exploring Methodological Approaches for Dynamic Connectivity in TMS–EEG

The present study focuses on the methodological aspects that involve the application of existing approaches in a novel context concerning the co-registration of TMS–EEG as a result of early visual area stimulation. We propose a TMS–EEG signal processing pipeline consisting of several phases: EEG preprocessing, TEP estimation, time-varying EC exploration, and graph network analysis. Finally, statistical tests were applied to extract and discern the significant differences between the stimulation sites.

Despite the robust methodology and clear results produced by this study, it is important to address the potential limitations associated with the experimental paradigm and analysis methodologies. Firstly, to estimate the TEPs, we employed a state-of-the-art Bayesian approach proposed by Sparacino et al. [23], which is particularly useful when the number of collected sweeps is limited and high variability is exhibited. In the field of TMS–EEG, the “ground truth” waveform of the TEP following occipital stimulation remains unknown, preventing a direct comparison between the Bayesian approach and the conventional one (i.e. CA) to demonstrate the superior performance of the former. However, the Bayesian approach has been validated on other datasets [24]. In our study, as shown in Figure 2, we observed significant differences between the potentials obtained with the Bayesian framework and the CA method. Secondly, despite the multi-level analysis involved in this study (TEP estimation, connectivity analysis with directionality, and time-varying graph analysis), it is important to note that our methodology does not require any manual parameter adjustment. One of the key strengths lies in the implementation of a Bayesian approach for TEP estimation, where the final prediction error (FPE) is used to determine the optimal order of the AR model. On the other hand, the estimation of the dynamic connectivity is performed using the STOK filter, which does not require any parameter selection, unless the optimal order of the AR model is once again estimated using the Akaike criterion. This automated approach ensures that the methodology is consistent and avoids potential biases that may arise from subjective parameter choices. In addition, the basal activity before each stimulation was subtracted. By applying this normalization, the different effects on brain connectivity after the stimulation can be compared among subjects. Despite this, a larger sample will be considered to enhance reproducibility.

## 5. Conclusions

To conclude, our study demonstrates the importance of connectivity and graph analysis measures in detecting hemispheric differences resulting from lateralized occipital TMS stimulation protocols. By capturing the time-varying dynamics of brain connectivity, we provided a more comprehensive understanding of how visual information is propagated across brain networks.

Compared with previous studies where the visual system was long considered equivalent across hemispheres, the observed electrophysiological patterns highlight hemispheric-specific effects as a consequence of the TMS stimulation. Knowing that the stimulation of specific brain areas can elicit visual percepts and how this stimulation spreads throughout the brain can provide a theoretical background for many practical and clinical applications. In conclusion, our study paves the way for further investigation in the field of visual area stimulation but other aspects need to be investigated. For example, having a “ground truth” electrophysiological response could help to better analyze the TEPs and related brain networks. Our future work will be focused on the integration of the inverse problem for EEG source localization to study the pattern of communication between the two human brain hemispheres for deep and superficial sources. 

## Data Availability

The data used in this study can be found at https://gin.g-node.org/DB_123/Phosphenes_occipital.git, accessed on 28 September 2023.

## Abbreviations

The following abbreviations are used in this manuscript:

TMS	transcranial magnetic stimulation
EEG	electroencephalography
TEP	TMS-evoked potential
M1	primary motor cortex
DLPFC	dorsolateral prefrontal cortex
MEP	motor-evoked potentials
SNR	signal-to-noise ratio
CA	conventional averaging
FC	functional connectivity
EC	effective connectivity
PLV	phase-lag value
PLI	phase-lag index
GC	Granger causality
VAR	vector autoregressive
DTF	directed transfer function
PDC	partial directed coherence
iPDC	information partial directed coherence
DCM	dynamic causal modeling
dFC	dynamic functional connectivity
fMRI	functional magnetic resonance imaging
PET	positron emission tomography
STOK	self-tuning optimized Kalman
PT	phosphene threshold
MOCS	method of constant stimuli
MSO	maximum stimulator output
ICA	independent component analysis
AR	autoregressive
tvMVAR	time-varying multivariate autoregressive
GMFP	global mean field power
BCT	brain connectivity toolbox
tdPLV	time-delayed phase locking value
FPE	final prediction error
AIC	Akaike’s information criterion
SVD	singular value decomposition

## Appendix A. Bayesian Framework to Estimate the TEPs

In this section, we present the state-of-the-art Bayesian approach used to obtain the unknown TEPs [23,24]. If *n* is the number of samples after a certain stimulus, we define the vectors as:

(A1) $$\begin{array}{c} \begin{array}{c} y={\left[y_{1},y_{2},\ldots ,y_{n}\right]}^{T}\\ u={\left[u_{1},u_{2},\ldots ,u_{n}\right]}^{T}\\ v={\left[v_{1},v_{2},\ldots ,v_{n}\right]}^{T} \end{array} \end{array}$$

where *y* is the observable EEG samples, *u* is the unknown TEP elicited in response to a stimulus, and *v* is the noise affecting *y*.

Using the observation model,

(A2) $$\begin{array}{c} y=u+v \end{array}$$

the aim is to recover *u* from *y* in Equation (A2). Assuming that *u* and *v* are uncorrelated zero-mean random vectors with “a priori” covariance matrices denominated by $\Sigma_{u}$ and $\Sigma_{v}$, we can obtain the linear mean square estimate of *u* given *y* as follows:

(A3) $$\begin{array}{c} \hat{u}={\left(\Sigma_{v}^{-1}+\Sigma_{u}^{-1}\right)}^{-1}\Sigma_{v}^{-1}y \end{array}$$

To solve Equation (A3), “a priori” covariance matrices $\Sigma_{v}$ and $\Sigma_{u}$ are required. The primary constituent of *v* is the EEG, which is widely recognized for its non-stationary nature. However, for brief intervals (e.g., 1–2 s), stationary AR models can be used for their description. Thus, $\Sigma_{v}$ can be obtained as follows:

(A4) $$\begin{array}{c} \Sigma_{v}=\sigma^{2}{\left(A^{T}A\right)}^{-1} \end{array}$$

where *A* is an *n*-dimensional Toeplitz matrix with its first column ${\left[1,a_{1},a_{2},\ldots ,a_{p},0,\ldots ,0\right]}^{T}$, $\left\{a_{k}\right\}$ representing the coefficients of the AR model of order *p*, and $\sigma^{2}$ is the variance of the white noise process that drives the AR model. It is possible to numerically determine $\left\{a_{k}\right\}$, *p*, and $\sigma^{2}$ before each stimulus using a least-square method, such as the Yule–Walker approach. The optimal AR model order, i.e., *p*, is selected according to an information criterion, for example, the FPE or Akaike’s information criterion (AIC) [74]. Considering the “a priori” known smoothness of the unknown TEP, it is possible to describe it as a realization of a stochastic process derived by the cascade of *d* integrators driven by a zero-mean white noise process with variance $\lambda^{2}$ [75]. This model can be described as

(A5) $$\begin{array}{c} \Sigma_{u}=\lambda^{2}{\left(F^{T}F\right)}^{-1} \end{array}$$

where = $F=\Delta^{d}$, with Δ representing an *n*-dimensional lower-triangular Toeplitz matrix with its first column ${\left[1,-1,0,\ldots ,0\right]}^{T}$.

By substituting $\Sigma_{v}$ and $\Sigma_{u}$ in Equation (A3), the equation is:

(A6) $$\begin{array}{c} \hat{u}={\left(A^{T}A+\gamma F^{T}F\right)}^{-1}A^{T}Ay \end{array}$$

where $\gamma =\frac{\sigma^{2}}{\lambda^{2}}$ is the unknown “smoothing parameter”. The Hall and Titterington criterion can be used to adjust the parameter γ until Equation (A7) is satisfied:

(A7) $$\begin{array}{c} WRSS\left(\gamma \right)=\sigma^{2}EDF\left(\gamma \right) \end{array}$$

where $WRSS\left(\gamma \right)={\left(y-\hat{u}\right)}^{T}A^{T}A\left(y-\hat{u}\right)$ denotes the weighted residuals sum of squares, and $EDF\left(\gamma \right)=n-trace[A^{T}{\left(A^{T}A+\gamma F^{T}F\right)}^{-1}A]$ represents the so-called equivalent degree of freedom. Once we have provided $\hat{u}$ and obtained the estimation error as $\tilde{u}=\hat{u}-u$, we obtained its covariance matrix, as a measure of the reliability of $\hat{u}$:

(A8) $$\begin{array}{c} cov\left(\tilde{u}\right)=\sigma^{2}{\left(A^{T}A+\gamma F^{T}F\right)}^{-1} \end{array}$$

If ${\hat{u}}^{i}$ is the *i*-th filtered sweep and ${\tilde{u}}^{i}$ is the corresponding estimation error, the estimate of the TEP is defined as follows:

(A9) $$\begin{array}{c} \bar{u}=\frac{\sum_{i=1}^{N}w_{i}{\hat{u}}^{i}}{\sum_{i=1}^{N}w_{i}} \end{array}$$

where $w_{i}$ are the weights given by the inverse of $trace[cov{\left(\tilde{u}\right)}^{i}]$.

## Appendix B. Self-Tuning Optimized Kalman (STOK) Filter

A tvMVAR model can be specified in the form of:

(A10) $$\begin{array}{c} Y_{t}=\sum_{k=1}^{p}A_{k,t}Y_{t-k}+\epsilon_{t} \end{array}$$

where $A_{k,t}$ are the AR matrices containing the time-varying model coefficients, $\epsilon_{t}$ is the zero mean white noise with covariance matrix $\Sigma_{\epsilon }$, and *p* is the model order. In general, the state-space model can be used to estimate the unknown variables $A_{k,t}$ and $\Sigma_{\epsilon }$, given by:

(A11) $$\begin{array}{c} \begin{array}{cc} x_{t} & =\Phi_{t-1}x_{t-1}+\omega_{t-1}\\ z_{t} & =H_{t}x_{t}+v_{t} \end{array} \end{array}$$

where $x_{t}$ is the system state, Φ is the transition matrix, $\omega_{t-1}$ is a zero-mean white noise of covariance *Q*, $z_{t}$ is the observed data, *H* is the projection measurement matrix, and $v_{t}$ is a zero-mean white noise (covariance *R*). The widely employed Kalman filter can effectively estimate the hidden state *x* and the error covariance at each time $\left(t=t_{1},\ldots ,t_{T}\right)$ by alternating between the prediction and the update step.

The state and the error covariance can be expressed as:

(A12) $$\begin{array}{c} \begin{array}{cc} \; & {\hat{x}}_{t}^{\left(-\right)}=\Phi_{t-1}{\hat{x}}_{t-1}^{\left(+\right)}\\ \; & P_{t}^{\left(-\right)}=\Phi_{t-1}P_{t-1}^{\left(+\right)}\Phi_{t-1}^{T}+Q_{t-1} \end{array} \end{array}$$

where ${\hat{x}}_{t}^{\left(-\right)}$ and $P_{t}^{\left(-\right)}$ are the "a priori" state and error covariance at time *t*, based on the previous estimated state and covariance ${\hat{x}}_{t-1}^{\left(+\right)}$ and $P_{t-1}^{\left(+\right)}$. A posteriori estimates of the state and error are refined in the update step, as:

(A13) $$\begin{array}{c} \begin{array}{cc} \; & K_{t}=P_{t}^{\left(-\right)}H_{t}^{T}{\left(H_{t}P_{t}^{\left(-\right)}H_{t}^{T}+R_{t}\right)}^{-1}\\ \; & {\hat{x}}_{t}^{\left(+\right)}={\hat{x}}_{t}^{\left(-\right)}+K_{t}\left(z_{t}-H_{t}{\hat{x}}_{t}^{\left(-\right)}\right)\\ \; & P_{t}^{\left(+\right)}=\left(I-K_{t}H_{t}\right)P_{t}^{\left(-\right)} \end{array} \end{array}$$

where *I* is the identity matrix and $K_{t}$ is the Kalman Gain matrix.

When dealing with neurophysiological time series, the Kalman filter’s performance may not be guaranteed due to the absence of a known transition matrix Φ and measurements *H*. Additionally, the covariance matrices *R* and *Q* are unknown.

As a result, the STOK filter was introduced, eliminating the need for explicit assumptions about the covariance matrices *R* and *Q*. In Pascucci et al., the $HPH^{T}\approx cR$ relationship was considered. In particular, the error covariance matrix *P*, projected onto the measurement space *H*, is a scaled version of the measurement noise covariance *R*, with *c* being a tuning parameter. This assumption allows for a redefinition of the Kalman gain as follows:

(A14) $$\begin{array}{c} \begin{array}{cc} K_{t} & =P_{t}^{\left(-\right)}H_{t}^{T}{\left(H_{t}P_{t}^{\left(-\right)}H_{t}^{T}+R_{t}\right)}^{-1}\\ \; & =H_{t}^{+}cR_{t}{\left(cR_{t}+R_{t}\right)}^{-1}\\ \; & =cH_{t}^{+}{\left(c+1\right)}^{-1}=\frac{c}{1+c}H_{t}^{+} \end{array} \end{array}$$

where the apex + stands for the Moore–Penrose pseudoinverse. The state update becomes

(A15) $$\begin{array}{c} \begin{array}{cc} {\hat{x}}_{t}^{\left(+\right)} & ={\hat{x}}_{t}^{\left(-\right)}+K_{t}\left(z_{t}-H_{t}{\hat{x}}_{t}^{\left(-\right)}\right)\\ \; & ={\hat{x}}_{t}^{\left(-\right)}+\frac{c}{1+c}H_{t}^{+}\left(z_{t}-H_{t}{\hat{x}}_{t}^{\left(-\right)}\right)\\ \; & =\frac{{\hat{x}}_{t}^{\left(-\right)}+cH_{t}^{+}z_{t}}{1+c} \end{array} \end{array}$$

where ${\hat{x}}_{t}^{\left(+\right)}$ is the weighted average of past predictions and the least-squares reconstruction. To prevent overfitting and reduce the model complexity, we use a singular value decomposition (SVD)-based method as described in [48].
